# Evaluating the online delivery of an autistic-led programme to support newly diagnosed or identified autistic adults

**DOI:** 10.1177/23969415231189608

**Published:** 2023-07-27

**Authors:** Laura Crane, Caroline Hearst, Maria Ashworth, Jade Davies

**Affiliations:** Centre for Research in Autism and Education, University College London, London, UK; AutAngel, Reading, UK; Centre for Research in Autism and Education, University College London, London, UK

**Keywords:** Post-diagnostic support, peer group, psychoeducation, telehealth, autistic adults

## Abstract

**Background & aims:**

Exploring Being Autistic is an autistic-led group-based programme providing psychoeducation and peer support to newly identified/diagnosed autistic adults. In 2020, due to social distancing measures implemented following the coronavirus pandemic, Exploring Being Autistic was adapted for online delivery. Here, we aimed to replicate previous research into the in-person delivery of Exploring Being Autistic, to determine whether similar results were obtained when the programme was delivered online. Further, we aimed to identify the unique opportunities and challenges that online delivery afforded.

**Methods:**

We used a community-based participatory research (CBPR) approach, whereby the autistic developer and facilitator of Exploring Being Autistic worked collaboratively with a team of academic researchers throughout the research process. Together, we evaluated two iterations of the online Exploring Being Autistic programme, involving 16 attendees. Attendees completed questionnaires before, during and after the programme. Attendees were also invited to participate in two post-programme (group or individual) interviews: one following the completion of the programme (time one) and another 6–8 months later (time two). Attendees were included in the research if they completed at least one questionnaire or interview. Data were analysed qualitatively, using reflexive thematic analysis.

**Results:**

Experiences of participating in the programme tended to be positive. Participants appreciated the autistic-led nature of the programme, found unity in the diversity of the group, and developed a positive and practical outlook as a result of the programme. Further analyses of our data revealed mixed views regarding the online delivery of the programme. Opportunities of online delivery were noted, such as this mode of participation reducing cognitive load, enabling the programme to be accessible to more participants, and fostering meaningful social connections among participants. However, technology and practical issues were felt to cause barriers, and some human aspects of participation were felt to be ‘lost in translation’ (e.g., in breakout groups).

**Conclusions:**

The online delivery of the Exploring Being Autistic programme yielded similar results to previous, in-person evaluations of the programme. While we identified positive aspects of online delivery, this mode did not entirely suit everyone's needs.

**Implications:**

From the current findings, we can make several recommendations to develop online support for autistic people. First, flexibility is key. To make support accessible and inclusive to a broad range of autistic people, the option for attendees to engage in-person, online or in hybrid formats should be considered. Second, if delivering support online, the use of breakout rooms should be carefully considered. While participants appreciated the opportunity to meet different people, some participants found the unpredictability and lack of scaffolding associated with breakout rooms challenging. To mitigate these challenges, groups could be pre-determined and shared with the attendees in advance (although consideration should be given to how the groups ‘fit’ together, and whether groupings should be changed at set intervals). Gentle warnings should also be given to those in breakout rooms, to alert them of the need to re-join the main group. Finally, support with technological aspects relating to engagement should be prioritised.

Learning that one is autistic in adulthood, through self-identification or formal diagnosis, is a potentially life-changing experience (Crompton et al., 2022). The identification/diagnostic process for autistic adults can be positive, fostering self-understanding and self-compassion (Leedham et al., 2020; Lewis, 2016). However, there is often a lack of emotional/practical support at this time (Crane et al., 2018; Jones et al., 2014), meaning that autistic adults may find it challenging to process what the diagnosis means for them (Crompton et al., 2022). There are ‘significant opportunities for improvement’ regarding post-diagnosis/identification supports for autistic adults (Wigham et al., 2022), with recent work focused on group-based programmes. For example, via interviews with 12 adults who received their autism diagnosis in adulthood, Crompton et al. (2022) found that peer support may be particularly useful; affording support not available via other channels (e.g., fostering authentic connections with like-minded others).

Exploring Being Autistic is an autistic-led group-based programme providing psychoeducation and peer support to newly identified/diagnosed autistic adults, as well as to adults who are questioning whether they might be autistic. Crane et al. (2021) evaluated two iterations of Exploring Being Autistic, via community-based participatory research (CBPR), whereby the autistic developer and facilitator of the programme (CH) worked collaboratively with a team of academic researchers (led by LC) at all stages of the research process (e.g., in developing the research questions, in designing the study methods, in making sense of the results, in writing reports, and in disseminating the findings). Despite the rarity of CBPR in the field of autism research (den Houting et al., 2021), such approaches ensure that research is scientifically sound, while also being relevant and beneficial to the autistic community (den Houting et al., 2021). Importantly, in the context of the present study, the work enabled the development of an evidence-based for autistic-led support and services; an area in which autistic involvement has historically been lacking (Milton, 2020).

The evaluation of Exploring Being Autistic by Crane et al. (2021) involved 16 attendees of the programme completing a pre-programme survey (to identify their motivations for taking part), as well as interviews at two time points (immediately after the programme, and again 6 months later; to elicit participants’ reflections on the programme and its impact over the short- and long-term). Encouragingly, the evaluation of Exploring Being Autistic indicated that the programme was well received. Attendees appreciated the autistic-led nature of the programme, with the facilitator's positive outlook and insider expertise providing a refreshing change from previous encounters with non-autistic professionals. Attendees also benefitted from the sense of belonging that came from meeting a diverse group of autistic people with whom they felt comfortable sharing experiences. Finally, attendees valued the practical support they received, which improved their general outlook on being autistic and provided practical strategies to address day-to-day challenges.

In 2020, due to social distancing measures implemented following the COVID-19 pandemic, Exploring Being Autistic was adapted for online delivery. It was unclear whether the Exploring Being Autistic would be successful when delivered online, since existing research on autistic people's satisfaction with online support is mixed. A study involving interviews with seven autistic adults and 12 caregivers of autistic adults reported satisfaction with telehealth delivery, noting that it was ‘surprisingly equivalent’ to in-person delivery (Harris et al., 2021, p. 1). Positive aspects noted in relation to telehealth delivery included increased comfort due to the removal of sensory stressors, as well as removing barriers to communication with professionals (Harris et al., 2021). Yet a sense of dissatisfaction with telehealth delivery versus in-person delivery has also been reported. For example, via in-depth qualitative work with autistic people and parents (n = 144), Pellicano et al. (2022) reported ‘intense dissatisfaction’ with online or telephone delivery of services that were previously delivered in person. Autistic adults whose services switched to telehealth delivery during the pandemic noted issues such as challenges in having private conversations with others in the vicinity, and they lamented how telehealth substitutions lacked ‘humanity’ (p. 923). This dissatisfaction was reported to have dire consequences, including disengagement with important services and supports.

In the current study, we evaluated two online iterations of the Exploring Being Autistic programme delivered during the COVID-19 pandemic. Our goal was to replicate our original research into this programme, to determine whether similar results were obtained. We also aimed to identify the feasibility of online delivery, by examining the unique opportunities and challenges this method of delivery afforded. Our specific research questions were:

1. Do the benefits/challenges of the online version of Exploring Being Autistic reflect the same benefits/challenges documented in the previous in-person evaluation of the programme?
2. What are the unique challenges and opportunities of the online delivery of Exploring Being Autistic?

## Method

### Design

The current study was conceived by the autistic developer of Exploring Being Autistic, CH, who approached the academic researchers (led by LC) about co-designing an evaluation of the online delivery of the programme. The team then collaborated as equal partners throughout the entire research process, drawing on the principles of CBPR (see Nicolaidis et al., 2011, for further details).

We used a combination of questionnaires and semi-structured interviews to answer our research questions. These methods were chosen by CH and LC, who felt that they would be most acceptable to autistic participants, and would best enable us to understand participants’ experiences of the programme. We were particularly keen to avoid the use of standardised measures to elicit feedback, given the well-documented issues that autistic participants face when completing such measures (see Nicolaidis et al., 2020, for an overview).

Two iterations of the programme were evaluated: iteration one (*n *= 8) took place between 30 September and 2 December 2020 and iteration two (*n *= 8) took place between 24 February and 28 April 2021.

### Participants

The online Exploring Being Autistic programme was advertised via mailouts and websites of the autistic-led organisations AutAngel and Autism Matters. All 17 programme attendees (9 from iteration 1, 8 from iteration 2) were then invited to participate in the research by being sent a link to information about the research and an associated consent form. Sixteen attendees subsequently consented to participate in the research across the two iterations, with one attendee (from iteration one) choosing not to take part. As per Table 1, most participants were women (*n *= 10, 62.5%), with a formal autism diagnosis (*n *= 10, 62.5%) given within the past 5 years. Remaining participants self-identified as autistic (*n *= 4, 25%) or were exploring whether they were autistic (*n *= 2, 12.5%). Participants were 30–79 years old (average = 49.2 years; SD = 9.62). Participants’ names have been replaced by pseudonyms throughout.

**Table 1.** Participant information.

Iteration	Participant	Gender identity	Age	Attended a previous support group?	Rating of previous support group	Sessions attended (out of 10)	Completed pre questionnaire	Completed mid-questionnaire	Completed post questionnaire	Completed post interview	Completed 6–8 month follow-up interview
Iteration 1	Andie	Gender Queer	30–39	✗	–	10	✓	✓	✓	✓	✓
	Ben	Male	30–39	✓	8	10	✓	✓	✓	✓	✓
	Celeste	Female				10	✗	✓	✓	✓	✓
	Donna	Female	50–59	✓	1	9	✓	✓	✓	✓	✓
	Edward	Male	50–59	✗	–	10	✓	✓	✓	✓	✗
	Felicity	Female	50–59	✗	–	10	✓	✓	✓	✓	✓
	Grainne	Female	50–59	✗	–	10	✓	✓	✓	✓	✗
	Heather	Female	30–39	✓	9		✓	✗	✗	✗	✗
Iteration 2	Martin	Male	50–59	✗	–	9	✓	✓	✓	✓	✗
	Nicola	Female	40–49	✗	–		✓	✓	✗	✓	✓
	Owen	Male	50–59	✗	–		✓	✓	✗	✓	✗
	Pippa	Female	40–49	✗	–	10	✓	✓	✓	✓	✓
	Quentin	Male	50–59	✗	–	9	✓	✓	✓	✓	✓
	Rhonda	Female	50–59	✗	–	10	✓	✓	✓	✓	✓
	Sheila	Female	70–79	✓	4	10	✓	✓	✓	✓	✗
	Tessa	Female	40–49	✓	7	8	✓	✓	✓	✗	✗

### Materials

#### Programme

Exploring Being Autistic aimed to support participants to: explore what being autistic means to them; explain their autistic identity to others, request appropriate accommodations and adapt some of their own behaviour; understand how autistic strengths can be capitalised on and how autistic challenges can be mitigated, and connect with a peer group. Exploring Being Autistic comprised 10 two-hour group sessions that took place virtually (via Zoom) over a 10-week period. Topics covered within the 10 sessions were diverse in scope and included diagnosis/identification, social communication, sensory issues, executive function, attention and mental health. For more details regarding the programme content, see Crane et al. (2021). Exploring Being Autistic was designed and led by an autistic facilitator (CH), and the sessions included both the provision of information about autism and (optional) role plays/discussions. There were no significant changes to the content of the programme when delivered online relative to when delivered in-person.

#### Questionnaires

Questionnaires were administered before, during and after the programme, as part of the facilitator's ongoing evaluation of the programme (as per funder requirements). Consent was sought from research participants for these completed questionnaires to be made available to the research team. Full copies of the surveys, along with participants’ full responses, are presented in the Supplemental Materials. Below, we provide a brief overview of the data from the questionnaires that was relevant to the research questions addressed in the current study:

Pre-programme Questionnaire. Pre-programme questionnaires gathered a range of demographic information including: age, gender identity, whether the participant had an autism diagnosis and, if so, when this diagnosis was received. The questionnaire also involved open-ended questions about experiences at previous autism support groups (including a rating of their usefulness on a scale from 1 = not at all useful, to 10 = extremely useful) and motivations for taking part in Exploring Being Autistic. Finally, participants were asked open-ended questions around whether they had received enough information about the programme in advance of starting the group sessions and, if not, what additional information they would have benefitted from.

Mid-Programme Questionnaire. The mid-programme questionnaire asked participants open-ended questions to elicit details of particularly positive and/or helpful aspects of the programme, as well as inviting them to offer any suggestions for improvements. Participants were also asked open-ended questions to elicit the most valuable, and most difficult aspects of the programme.

Post-Programme Questionnaire. In the post-programme questionnaire, participants were asked how many sessions they attended (max = 10). They were then asked open-ended questions to probe for the most beneficial aspect of the programme, the session they found most helpful, and any suggestions for improvements. Participants could also provide an overall rating for the programme (where 1 was the lowest score, and 10 was the highest) and explain this rating, as well as stating whether they would recommend the programme to others.

#### Interviews

Interviews followed the structure and content reported in Crane et al. (2021), with additional open questions about the benefits and challenges of virtual delivery of the programme. Specifically, time one interviews, conducted immediately after the programme ended, covered the following topics: previous attendance at in-person or online support groups (probing for positive and negative aspects of these groups); motivations for joining the group and what they had hoped to gain; whether the programme met their expectations; and their overall appraisal of the programme (what worked well, and what could have been improved, particularly with reference to the online delivery). Interviews concluded with a discussion of the autistic-led nature of the programme, and participants were offered the opportunity to add any further thoughts or ask any questions (especially with reference to the online delivery of the programme).

Time two interviews, conducted 6–8 months after the programme ended, focused on participants’ reflections on the programme (with the benefit of hindsight). The following topics were covered: whether the participant was pleased that they took part in the programme; if/how they felt the programme affected them; aspects of the programme that they thought were helpful and unhelpful (particularly in relation to the online delivery of the programme); potential content or topics of discussion that the programme could have usefully covered; and whether participants had attended any support groups since the programme ended. As a final discussion point, the interviewer asked whether the participant had kept in contact with any members of the group. Full copies of the interview schedules are in the Supplemental Materials.

As per the suggestion of CH, the developer and facilitator of Exploring Being Autistic, participants were given the choice of taking part in a group or individual interview with LC. For those who opted for a group interview, CH (who had knowledge of the participants) provided guidance as to who might complement each other in a group interview and/or who would feel most comfortable together. Most interviews were conducted synchronously via Zoom, but one participant participated asynchronously via email.

### Procedure

Ethical approval for the research was obtained from the Research Ethics Committee at IOE, University College London's Faculty of Education and Society. The project was a collaboration between LC (a non-autistic autism researcher) and CH (the autistic developer and facilitator of the programme), with input from MA and JD (both non-autistic autism researchers). LC attended the final session of each iteration of the programme to introduce herself, the research, and answer any questions. LC also provided participants with a summary about her professional background and research interests, as well as the interview questions. This information was provided alongside a link to an online information sheet about the research, as well as an online consent form for participants who wanted to be involved. Participation in the programme was not contingent on research participation.

Quantitative data are presented descriptively. Qualitative data (from questionnaires and interviews) were analysed using reflexive thematic analysis (as detailed in Crane et al., 2021, based on Braun & Clarke, 2019). To answer research question one (do the benefits/challenges of the online version of Exploring Being Autistic reflect the same benefits/challenges documented in the previous in-person evaluation of the programme?), data were analysed using a deductive approach. Specifically, we approached our analysis with preconceived themes that we expected to find based on previous knowledge (i.e., based on our analysis of the in-person Exploring Being Autistic evaluation). The analytic process involved MA coding participants’ motivations to attend the course into the framework identified in Crane et al. (2021), whilst also examining whether any additional motivations could be identified. A second coder, JD, reviewed the transcripts and the suggested codes, confirming agreement with the other coder through recursive discussions. A similar approach was used for the interview data, except that both authors independently coded transcripts within the broader framework of themes identified in Crane et al. (2021); checking for additional themes, and engaging in recursive discussions to confirm agreement. During this process, one coder reviewed and coded transcripts from iteration one, while the other coder reviewed and coded transcripts from iteration two (with regular input from LC); drawing these codes together at the stage of theme development.

To answer research question two (what are the unique challenges and opportunities of online delivery of Exploring Being Autistic?), data analyses involved recursively proceeding through the stages of data familiarisation, data coding, theme development, and review (as per Braun & Clarke, 2019). Our analyses focused on identifying semantic and latent meaning in the dataset, following an inductive approach, whereby the analyses were approached without trying to fit our data within a pre-existing coding frame (Braun & Clarke, 2019). The analytic process was led by MA and JD, who independently coded data from one iteration of the programme, before engaging in discussion to agree initial codes and themes (in collaboration with LC). This process was repeated when data from the second iteration of the programme was collected. However, during the process of data familiarisation and data coding, it became apparent that codes and themes from the two iterations overlapped. As such, data across the two iterations were analysed and presented together.

To ensure the rigour of our analyses, we followed Nowell et al.'s (2017) recommendations, including: ensuring credibility (e.g., through prolonged engaged with the data, alongside member checking); ensuring transferability (e.g., through providing thick descriptions to enable others to judge the transferability of findings); ensuring dependability (e.g., through ensuring our process was logical, traceable and clearly documented); ensuring confirmability (e.g., through clearly stating our methodological and analytical choices); keeping clear audit trails (e.g., with individual coders keeping records of their analytical process, enabling analyses across coders to be related and cross-referenced); and engaging in reflexive practices (e.g., through members of the research team reflecting on the personal values, interests and experiences that they bring to the analytic process via peer discussions). In terms of our positonality, all authors were involved in the previous evaluation of Exploring Being Autistic, and came to the current analyses with a belief in the utility of the programme for the autistic community. As such, coders were careful to review data for both positive and negative reflections from participants. Further, all authors align with approaches to support for autistic people that are grounded in the social model of disability and are aligned with the concept of neurodiversity (see Pellicano & den Houting, 2022). Throughout our process of analysis, coders engaged in reflexive discussions to critically interrogate how our beliefs influenced, and contributed to, the research process.

## Results

### Research question 1: does the online version of Exploring Being Autistic yield the same benefits/challenges as outlined in the previous, in-person evaluation of the programme?

Data collected from the questionnaires and interviews revealed that motivations for enrolling in the programme largely mapped onto the themes specified in Crane et al. (2021). Specifically, theme 1 showed participants ***appreciated the autistic-led nature*** of the programme (‘Being in an autistic-led group is key, for me. It's invaluable, a relief, and has increased my confidence as no other situation has ever been able to’; Sheila) and theme 2 showed that participants ***found unity in the diversity*** of the group (‘it's just nice to see sort of diversity within the group and not being alone’; Donna). The third theme showed participants also ***developed a positive and practical outlook*** as a result of the programme (‘I don’t need to blame myself…we can just be the way we are…that's a much…healthier approach to life’; Celeste). Unique to the current study, however, was that some participants were motivated to take part after reading about the previous success of the programme: ‘I read the previous publication about it and that was actually what got me to join because I read the feedback and the responses…it seemed perfect’ (Andie).

### Research question two: what are the unique challenges and opportunities of the online delivery of Exploring Being Autistic?

**Figure 1.**
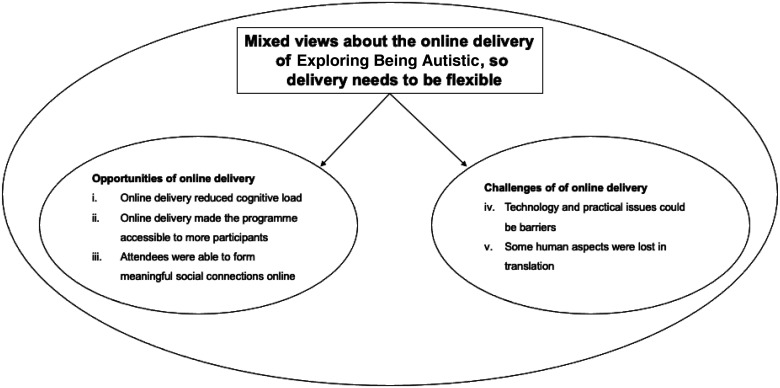
Thematic map.

Data collected from the interviews revealed an overall theme that there were ***mixed views about the online delivery of Exploring Being Autistic, so delivery needs to be flexible***. Within this overarching theme, five subthemes were identified. For purposes of readability, we organised the sub-themes to reflect how the delivery of Exploring Being Autistic offered both opportunities as well as challenges (see Figure 1).

#### Opportunities of online delivery

Sub-theme 1: Reducing cognitive load. Attendees explained how the online delivery of Exploring Being Autistic allowed them to preserve energy that would have otherwise been expended on factors associated with attending in-person events, such as travelling (‘finding places…being on the tube and the noise on the streets…all of that takes a sort of cumulative toll’; Andie), the sensory environment (‘you don’t get any additional noise [online] whereas you might do it in a hall’; Felicity) and ‘draining’ social interaction. Some participants spoke of the cost-benefit analysis involved in deciding to attend groups like Exploring Being Autistic, with online delivery removing many perceived ‘costs’: ‘my batteries aren’t already half drained from getting to the thing and all the other stuff’ (Ben). Being in a familiar environment also facilitated openness and improved engagement: ‘whenever a group is together face-to-face in a room there are additional peer-pressures which I find harder to be open…it's easier to join [in] online’ (Martin).

Sub-theme 2: Enhancing accessibility. Participants noted that online delivery removed travel-related barriers, allowing ‘others from greater distance to take part’ (Martin), as well as those who may struggle with the demands associated with travelling (‘to get into [town] on a weekly basis is not something that I would do’; Andie). Similarly, online delivery supported alternative ways of communicating: ‘you can interpret what people are thinking and feeling through what they’re writing…they might say only joking…[or use] emojis…it's easier’ (Donna).

Sub-theme 3: Forming meaningful social connections. Participants reflected that online delivery facilitated social interaction between attendees, providing ‘a kind of structure’ (Owen) and removing social pressures associated with in-person events. Breakout rooms were perceived as particularly useful; affording a unique level of privacy, and allowing attendees to ‘discuss things which you wouldn’t want to talk about in a larger group’ (Edward). Participants also appreciated how breakout rooms facilitated socialisation with different attendees (‘I got to be with different people each time’; Andie) and helped remove the ‘uncertainty of when is it my turn to talk’ (Andie). Some connections made during the programme had been sustained, with one participant reporting that relationships had since strengthened: ‘We do still have that rapport, if anything, it's grown a little bit among the ones of us that have stayed in touch’ (Pippa).

#### Challenges of online delivery

Sub-theme 4: Technology and practical issues could be barriers. Online delivery yielded unique technological challenges. Without the course facilitator present in breakout rooms, one participant suggested there ‘wasn’t enough scaffolding’ (Ben) which left some participants ‘a little bit confused about what to do’ (Nicola). The unpredictability of the breakout rooms was also ‘a bit difficult…because you didn’t know who was gonna be [there]’ (Martin). The transition between breakout rooms and the main group could ‘feel quite abrupt’ (Celeste), although others noted that ‘[the facilitator sent a] warning saying two minutes to the end, so you could wind it down’ (Donna).

Participants reflected that the group spent ‘quite a bit of time with technical issues’ (Ben), causing delays. Individual challenges with technology (e.g., ‘bad [internet] connections’; Andie) and difficulties using multiple platforms and technologies were noted: ‘[you had to] go from the email…to [the forum] and then you had to download this worksheet…and then I thought I need to print…[but the printer was] low on ink’ (Rhonda). Some attendees also experienced *Zoom fatigue*,^1^ finding the process ‘intense’ (Ben) and ‘tiring’ (Grainne). Importantly, however, such issues ‘didn’t impinge too much in the enjoyment of the programme’ (Nicola).

Sub-theme 5: Some human aspects were lost in translation. Some participants felt the online format lost a degree of sensitivity. For example, one participant explained ‘in the online format if someone gets upset…and leaves, or the session comes to an end, they are…left…quite triggered’ (Ben). Indeed, another participant reported that ‘[the facilitator] couldn’t see that I was becoming upset…[because] the pictures on the screen were so small’ (Grainne). Online delivery also added ‘an extra level of difficulty’ (Grainne) to the social aspect of the course, with one participant explaining ‘trust and confidence took a little bit longer [to build] than it would have done…face-to-face’ (Pippa). Indeed, there were fewer opportunities ‘to form a more personal, social connection…[and] find common ground besides being autistic’ (Andie), as many participants would ‘turn off their screens’ during breaks (Andie). Consequently, participants missed the spontaneous conversations they would have in-person: ‘people go and make themselves a coffee [and] you chat there to somebody for a couple of minutes and then you talk to somebody else when you’re sitting back down again, [that works] better in-person’ (Celeste).

Overall, and taking the advantages and challenges into account, participants noted ‘[there is a] need for flexibility and offering different ways for people to engage with the programme’ (Martin).

## Discussion

The results of this study were encouraging: the online delivery of Exploring Being Autistic yielded similar outcomes to the previous in-person delivery (Crane et al., 2021). For example, participants appreciated the autistic-led nature of the programme, found unity in the diversity of the group, and also developed a positive and practical outlook following their participation. Yet specific consideration of the online delivery of the course highlighted several unique opportunities (e.g., reducing cognitive load, enhancing accessibility, fostering meaningful social connections), and challenges (e.g., technology and practical barriers, some human aspects being lost online). Participants concluded that the delivery of future iterations must be flexible, accounting for individual preferences.

It should be emphasised that the online delivery of Exploring Being Autistic, evaluated here, took place during an unprecedented and unique context (the coronavirus pandemic), which may have influenced participants’ perceptions and engagement with the course. The unique and significant impact of the pandemic on autistic people included increased uncertainty, changes to routine, and social isolation, which affected autistic people's mental health (see Scheeran et al., 2023). Engaging in a peer support group during this time might have been especially beneficial as it provided a regular, structured opportunity for attendees to engage with other autistic people. Additionally, these iterations of Exploring Being Autistic were delivered at a time when the course facilitator and attendees were becoming more accustomed to online formats. As such, participants’ evaluation of technical issues and the unfamiliarity of the online format may be less applicable to future iterations.

Attendees’ mixed views about the various opportunities and challenges of online delivery aligns with emerging research about remote support for autistic people. For instance, attendees felt that online delivery was accessible and reduced cognitive load as they could participate from familiar, comfortable environments and did not have to expend energy on travelling or other social stressors (e.g., Harris et al., 2021). However, some attendees found it more difficult to socialise online, particularly given the use of breakout groups (which were felt to lack scaffolding without the facilitator present, and could be abrupt in the way that they ended). This finding aligns with the results of a study by Pellicano et al. (2022), whose autistic participants found online interactions to be ‘a poor substitute for real interaction’.

From the current findings, we can make several recommendations to develop online support for autistic people. First, flexibility is key. To make support accessible and inclusive to a broad range of autistic people, the option for attendees to engage in-person, online, or in hybrid formats should be considered. Second, if delivering support online, the use of breakout rooms should be carefully considered. While participants appreciated the opportunity to meet different people, some participants found the unpredictability and lack of scaffolding associated with breakout rooms challenging. To mitigate these challenges, groups could be pre-determined and shared with the attendees in advance (although consideration should be given to how the groups ‘fit’ together, and whether groupings should be changed at set intervals). Gentle warnings should also be given to those in breakout rooms, to alert them of the need to re-join the main group. Breakout rooms could also be used for informal socialisation, if desired, to help foster more social connections. Finally, support with technological aspects relating to engagement should be prioritised.

### Limitations

There were several limitations of this study. First, not all attendees took part in the 6–8 month follow-up interviews, meaning there may have been a self-selection bias whereby those with more negative or apathetic view of the course did not contribute. That said, the results represent positive and negative insights about the course. Second, while we adopted participatory principles in our research (this project was conceived by, and co-designed with, the autistic creator of the programme) we did not have autistic involvement at every stage of the research (specifically, non-autistic researchers led data analysis). A copy of the full report was, however, shared with our autistic participants who were invited to comment on the results and recommendations.

### Conclusions

To conclude, the current study confirmed that the online delivery of the Exploring Being Autistic programme yielded similar results to previous, in-person evaluations of the programme. While we identified positive aspects of online delivery, this mode did not entirely suit everyone's needs. Participants suggested that the delivery of support for autistic people must be flexible to be truly accessible to different people's needs and preferences.

## Supplemental Material

sj-docx-1-dli-10.1177_23969415231189608 - Supplemental material for Evaluating the online delivery of an autistic-led programme to support newly diagnosed or identified autistic adults Click here for additional data file. Supplemental material, sj-docx-1-dli-10.1177_23969415231189608 for Evaluating the online delivery of an autistic-led programme to support newly diagnosed or identified autistic adults by Laura Crane, Caroline Hearst, Maria Ashworth and Jade Davies in Autism & Developmental Language Impairments

## Notes

1. A phenomenon whereby repeated video conferencing meetings can cause people to become more exhausted compared to face-to-face meetings (Shoshan & Wehrt, 2022).
