# Supplementary material for: Evaluating the online delivery of an autistic-led programme to support newly diagnosed or identified autistic adults
Source: Autism Dev Lang Impair. 2023 Jul 27;8:23969415231189608. doi: 10.1177/23969415231189608 (PMC10387681; doi:10.1177/23969415231189608)
Supplement: sj-docx-1-dli-10.1177_23969415231189608 - Supplemental material for Evaluating the online delivery of an autistic-led programme to support newly diagnosed or identified autistic adults [file sj-docx-1-dli-10.1177_23969415231189608.docx]

**Appendix A**

**[XXX] Pre-programme Questionnaire**

***Part One: About You and Previous Support***

Name (optional) __________

What is your age? __________

What is your gender? _________

Do you have an autism diagnosis. Yes / No (delete as appropriate)

If so when did you get the diagnosis? __________

Have you attended any autism support groups or programmes in the past? If yes, please rate the usefulness of the group [note: if you have attended more than one programme in the past, please rate the most recent group you attended]

| 1 | 2 | 3 | 4 | 5 | 6 | 7 | 8 | 9 | 10 |
| --- | --- | --- | --- | --- | --- | --- | --- | --- | --- |
| Not at all  useful | |  |  |  |  |  |  |  | Extremely useful |

We would love to hear more about this, if you were willing to share:

***Part Two: About your participation in this programme***

Please tell us why you wanted to take part in this programme:

What do you hope to gain from taking part in this programme?

Do you feel that you have received enough information about the nature/content/purpose of the programme, prior to starting the group sessions?

Yes No

If no please explain what you would have liked to know:

**[XXX] Mid-programme Questionnaire**

| Was information presented in a logical, easy to understand way? Circle answer below |
| --- |
| the pacing of the trainer’s delivery was too slow just right too fast |
| The amount of material covered was too little just right too much |
| The amount of interaction was too little just right too much |

What have you found positive and/or helpful about the group so far?

Is there anything about the group you feel is negative or unhelpful for you?

Do you have any suggestions about how the group could be changed to make it better for you?

What has been the most valuable thing about the group so far?

What has been the most difficult thing about the group so far? (For example are there could be negative feelings evoked by the thinks people say that you don’t feel you have time to explore in the group so you leave feeling bad)

**[XXX] Post-programme Questionnaire**

How many sessions did you attend? __________

What was the most beneficial aspect of the programme, and why?

Which session (if any) did you find most helpful, and why?

What changes (if any) could be made to improve the programme?

Please provide an overall rating for the programme, where 1 is the lowest score and 10 is the highest score:

| 1 | 2 | 3 | 4 | 5 | 6 | 7 | 8 | 9 | 10 |
| --- | --- | --- | --- | --- | --- | --- | --- | --- | --- |

You may explain your answer below, if you wish:

Would you recommend this programme to other people? Yes / No

|  | Tick if it applies to you. |
| --- | --- |
| Participants attending the course and will meet other autistic adults and learn about autism |  |
| Participants better understand how autism affects them and learn strategies to help them cope. They join a supportive peer community. |  |
| Participants will feel less isolated and lonely. |  |
| Participants will be able to identify when work related problems are connected to autism. |  |
| Those experiencing difficulties at work will have the support of the community to help them address their difficulties with their employers. |  |
| Participants will be happier in their workplace, more understood by co-workers and better able to maintain employment. |  |
| Participants will meet other autistic adults. |  |
| Participants will make friendships and have opportunities to access other social groups through connections within the groups. |  |

**Appendix B. Interview Guide**

**Time One interview**

| **Introduction** | [XXX] will remind you about some key information, such as:   - Why we’re doing the research - That the interview will be recorded, if it’s ok with you - That you don’t have to answer any questions if you don’t want to - What will happen to the information you provide |
| --- | --- |
| **About you** | [XXX] will ask you about:   - Whether you have a formal autism diagnosis or self-identify as autistic - When this was |
| **Your experience of previous autism groups** | [XXX] will ask whether you’ve taken part in any autism-related support groups/programmes in the past:   - If so, what was the structure (e.g., autistic-led, online/face-to-face) and what did you think of them? - If not, why haven’t you attended any? |
| **About** [XXX] **– motivations for attending** | [XXX] will ask:   - Why you wanted to take part in the programme - Whether the online format influenced your decision to take part - Whether your expectations were met |
| **About** [XXX] **– the programme itself** | [XXX] will ask for your views on:   - The format of the programme - The most helpful things about the programme - Aspects of the programme that could be improved in future - Your views on the autistic-led nature of the programme |
| **Anything else?** | [XXX] will ask if you have any extra comments you would like to share about the programme. |
| **End of interview** | You can ask any questions you have about the research (if you have any) or we can end the interview. |

**Time two interview**

| **Introduction** | [XXX] will remind you about some key information, such as:   - Why we’re doing the research - That the interview will be recorded, if it’s ok with you - That you don’t have to answer any questions if you don’t want to - What will happen to the information you provide |
| --- | --- |
| **Question 1** | [XXX] will ask:  On reflection – six months since completing the course - are you pleased that you took part in the programme? |
| **Question 2** | [XXX] will ask:  Are there any specific aspects of the programme that you have found particularly useful/helpful in the time since the programme ended? |
| **Question 3** | [XXX] will ask:  Is there anything you learned about or practised in the programme that you haven’t found helpful/useful? |
| **Question 4** | [XXX] will ask:  The programme was a ten-week programme – have you taken part in any support groups or programmes since? Were these online or face-to-face? |
| **Question 5** | [XXX] will ask:  Have you maintained contact with the group members? |
| **End of interview** | You can ask any questions you have about the research (if you have any) or we can end the interview. |

**Appendix C. Evaluation of the** [XXX] **programme**

***Quantitative data: mid-programme questionnaire***

|  | **Too slow** | **Just right** | **Too fast** |
| --- | --- | --- | --- |
| **Pacing of the delivery of the programme (*n* = 13)** | 1 (7.7%) | 11 (84.6%) | 1 (7.7%) |
|  | **Too little** | **Just right** | **Too much** |
| **Amount of material covered in the programme (*n* = 12)** | 2 (16.7%) | 10 (83.3%) | 0 (0%) |
| **Amount of interaction during the programme (*n* = 13)** | 1 (7.7%) | 12 (92.3%) | 0 (0%) |

***Quantitative data: post-programme questionnaire***

| **Overall rating of the programme (max = 10)** | Mean = 9, SD = 0.82 | | |
| --- | --- | --- | --- |
|  | **Yes** | **No** | **Maybe** |
| **Would you recommend this programme to other people?** | 13 (100%) | 0 (0%) | (0%) |
| **Participants attending the course and will meet other autistic adults and learn about autism** | 12 (92.3%) | 1 (7.7%) | (0%) |
| **Participants better understand how autism affects them and learn strategies to help them cope. They join a supportive peer community.** | 12 (92.3%) | 1 (7.7%) | (0%) |
| **Participants will feel less isolated and lonely.** | 10 (76.9%) | 3 (15.4%) | (0%) |
| **Participants will be able to identify when work related problems are connected to autism.** | 8 (61.5%) | 5 (38.5%) | (0%) |
| **Those experiencing difficulties at work will have the support of the community to help them address their difficulties with their employers.** | 5 (38.5%) | 8 (61.5%) | (0%) |
| **Participants will be happier in their workplace, more understood by co-workers and better able to maintain employment.** | 2 (15.4%) | 11 (84.6%) | (0%) |
| **Participants will meet other autistic adults.** | 11 (84.6%) | 2 (15.4%) | (0%) |
| **Participants will make friendships and have opportunities to access other social groups through connections within the groups.** | 9 (69.2%) | 2 (15.4%) | 2 (15.4%) |
